# miR-25 Regulates Gastric Cancer Cell Growth and Apoptosis by Targeting EGR2

**DOI:** 10.3389/fgene.2021.690196

**Published:** 2021-10-26

**Authors:** Liuqing Yang, Lina Li, Pan Chang, Ming Wei, Jianting Chen, Chaofan Zhu, Jing Jia

**Affiliations:** ^1^ Second Affiliated Hospital of Xi’an Medical University, Xi’ an, China; ^2^ First Department of Medical Oncology, Affiliated Shaanxi Provincial Cancer Hospital, Xi’an, China; ^3^ Department of Pharmacology, Xi’an Medical University, Xi’an, China

**Keywords:** gastric cancer, MiR-25, cell growth, apoptosis, Egr2

## Abstract

Gastric cancer is one of the most common malignancies harmful to human health. The search for effective drugs or gene therapy has aroused the attention of scientists. So far, microRNAs, as small non-coding RNAs, have the potential to be therapeutic targets for cancer. Herein, we found a highly expressed miR-25 in gastric cancer cell. However, the function of miR-25 for gastric cancer cell growth and apoptosis was unknown. Functionally, we used RT-qPCR, western blot, CCK-8, and flow cytometry to detect gastric cancer cell growth and apoptosis. The results indicated that miR-25 promoted gastric cancer cell growth and inhibited their apoptosis. Mechanistically, we found that a gene EGR2 was a potential target gene of miR-25. Further dual-luciferase results supported this prediction. Moreover, knockdown of EGR2 promoted gastric cancer cell growth and inhibited their apoptosis by flow cytometry detection. Altogether, these findings revealed miR-25 as a regulator of gastric cancer cell growth and apoptosis through targeting EGR2.

## Introduction

Gastric cancer is one of the most common malignancies of the digestive system, which endangers human life and health (Bertuccio et al., 2009; Ferlay et al., 2015). In the last few decades, many achievements have been made in gastric cancer -related proto-oncogenes, suppressor genes, and related signal transduction pathways, but the exact mechanism of tumorigenesis has not been fully elucidated. The emergence of a new class of non-protein coding microRNA (miRNA) provides a new idea for gastric cancer research. MiRNAs play an important role in the pathogenesis of tumors, which provides a potential new strategy for understanding the clinical therapeutics of gastric cancer (Li et al., 2011). MiRNAs generally bind to their target mRNAs through complementary pairing, thus regulating the expression of target genes at the post-transcriptional level (Brennecke et al., 2003).

Recent studies have shown that miRNA plays an important role in the pathogenesis and progression of gastric cancer, which provides us with a potential direction for the study of therapeutic targets for this major disease (Huang et al., 2016; Zhang et al., 2019). Studies have shown that abnormal expression of miRNAs plays a crucial role in the pathogenesis and malignant biological phenotypes of gastric cancer, such as proliferation, apoptosis, invasion, and migration of gastric cancer cells (Wang et al., 2016; Zhang et al., 2016; Zhang et al., 2017a; Zhang et al., 2017b). MiR-25 is widely expressed in various types of tumors, and previous studies have shown that its expression level is related to the clinical characteristics and prognosis of tumors (Xu et al., 2014; Mingjun, 2015; Peng et al., 2015; Wang et al., 2015). However, the role of miR-25 in different tumors is consistent but the target genes are different (Caiazza and Mallardo, 2018). Studies have reported that miR-25 plays an oncogene role in lung cancer (Wu et al., 2015), liver cancer (Wang et al., 2015; Tian and Yao, 2016; Avencia et al., 2019), and gastric cancer (Li et al., 2015). MiRNA-25 had been investigated in gastric cancer via targeting RECK (Zhao et al., 2014), but we found that there might be other targets. Therefore, in this study, we explored the biological effect and significance of miR-25 in the tumorigenesis of gastric cancer, looking for new targets and hoping to provide a new experimental basis for understanding the pathogenesis and therapeutics of gastric cancer.

In this study, we detected the expression feature of miR-25 in normal gastric epithelial cells and gastric cancer cells by RT-qPCR. The result revealed that the expression level of miR-25 in gastric cancer cells was significantly higher than that in normal cells. Overexpression of miR-25 promoted gastric cancer cells’ growth and inhibited their apoptosis. Mechanically, we demonstrated that *EGR2* was a target gene of miR-25 by dual-luciferase reporter gene assay in gastric cancer cells.

## Material and Method

### Cell Culture

The cell line GES-1 cells (ATCC) and BGC823 cells (ATCC) were preserved in this laboratory. The cells were placed in DMEM medium containing 10% fetal bovine serum, 1 × 10^5^ IU/L penicillin, and 1 × 10^5^ IU/L streptomycin conventional culture in an incubator at 37°C and 5% CO_2_.

### Cell Transfection

One day before transfection, cells were transferred to a suitable density so that the cells were in a logarithmic growth phase at the time of transfection. Firstly, DMEM was used to culture cells for about 4 h excluding serum and antibiotics. Then, negative control, miRNA mimic or inhibitor, and siRNA were transfected into cell by Lipofectamine 2000 (Invitrogen) according to operation instructions. After transfection for 6 h, the fresh medium was replaced and cultured for a continuous 48 h for further experiments.

### Real Time Quantitative PCR

The total RNA was extracted with Trizol kit, and the concentration of RNA was determined by Nucleic acid analyzer. The first strand cDNA of miRNA was synthesized by reverse transcription reaction of stem-loop primers. The SYBR GREEN reagent was used for PCR amplification according to kit instructions. The real-time quantitative PCR (RT-qPCR) reaction was performed on an ABI PCR instrument and the results were analyzed. All primer sequences were listed in Table 1.

**TABLE 1:** The gene primer sequence for RT-qPCR or PCR.

Gene name	Forward primer	Reverse primer
miR-25 RT	GTC​GTA​TCC​AGT​GCA​GGG​TCC​GAG​GTA​TTC​GCA​CTG​GAT​ACG​ACT​CAG​AC	
miR-25	AGTCTGGCTCTGTTCACG	CCA​GTG​CAG​GGT​CCG​AGG​TA
Bax	CCC​GAG​AGG​TCT​TTT​TCC​GAG	CCA​GCC​CAT​GAT​GGT​TCT​GAT
p53	CAG​CAC​ATG​ACG​GAG​GTT​GT	TCA​TCC​AAA​TAC​TCC​ACA​CGC
Bcl-2	GGT​GGG​GTC​ATG​TGT​GTG​G	CGG​TTC​AGG​TAC​TCA​GTC​ATC​C
EGR2	TCA​ACA​TTG​ACA​TGA​CTG​GAG​AG	AGT​GAA​GGT​CTG​GTT​TCT​AGG​T
EGR2 (3′UTR wild)	GCTC​GAGGAC​CCT​GGA​TGT​CAG​AGT​TG	GGCG​GCC​GCCCT​CTG​AGA​TCA​TAA​ATG​C
EGR2 (3′UTR mutant)	CAG​AGA​ACA​GAA​GGT​CCA​TGT​GAT​GGG	CCC​ATC​ACA​TGG​ACC​TTC​TGT​TCT​CTG

### Western Blot

Proteins of each group were extracted and quantified with BCA protein quantitative kit. After SDS-PAGE, the proteins were transferred to PVDF membrane by electrol transfer. The product was sealed with 5% skim milk at room temperature for 60 min, separately added with corresponding primary antibody, incubated at 4°C overnight, and washed with TBST three times. Then, the secondary antibody was added at room temperature for 2 h incubation, and then washed with TBST three times. After exposure in the darkroom, the results were processed by Quantity One gel analysis software. Primary antibody Bax (21kDa, source: mouse), Bcl-2(26kDa, source: mouse) and β-actin (42kDa, source: mouse) were purchased from Proteintech. β-actin acts as the internal gene.

### CCK-8 Assay

About 5 × 10^4^ /ml cell suspensions were prepared. 100 μl cell suspension was added to the 96-well plate and continued to be cultured in an incubator at 37°C. At different time points, 10 μl CCK-8 solution was added to each well and incubated at 37°C for 1–4 h. The absorbance value at 450 nm was determined.

### Cell Cycle Assay

To detect the cell cycle assay, gastric cancer cells were fixed with 70% ethanol for about 24 h at −20°C. The cells were labeled with propidium iodide (PI) for 30 min at 37°C and detected in the BD FACSVerse flow cytometer. The results of cell cycle were analyzed using the cell cycle analysis software. The detailed experimental process follows the manufacturer’s instructions (Multisciences, Hangzhou, China).

### Cell Apoptosis Assay

The cells were collected directly into a 10 ml centrifuge tube. The rinse solution was washed once, and centrifuged at 800 r/min for 5 min. The cells were resuspended in solution and incubated in the dark for 15 min at room temperature. The precipitated cells were centrifuged at 800 r/min for 5 min and incubated with buffer solution once. The fluorescent dye solution was added and incubated at 4°C for 20 min to avoid light. The wavelength of excitation light in the flow cytometer was 488 nm, the wavelength of FITC was detected by a 515 nm passband filter, and the other wave length was greater than 560 nm to detect PI. On the scatter diagram of bivariate flow cytometry, living cells are shown in the lower left quadrant, which is (FITC-/PI-). The upper right quadrant is the non-living cells, i.e., dead cells, which are (FITC +/PI +). The lower right quadrant for apoptotic cells is (FITC +/PI-).

### Luciferase Report Assay

The target genes predictions were revealed by bioinformatics software Targetscan 7.2 (http://www.targetscan.org/vert_72/) online. The wild-type 3′UTR or mutant-type 3′UTR of EGR2 mRNA sequence were inserted into luciferase reporter plasmids psi-CHECK2™ (Promega). EGR2 wild-type or mutant-type luciferase reporter vector and miR-25 mimic or negative control were co-transfected into 293T cells. Following the manufacturer’s instruction of Dual Luciferase Assay System (Promega), luciferase activities were measured.

### Statistics Analysis

All data were expressed as mean ± standard deviation. SPSS18.0 software was used for statistical analysis, and student’ t-test was used for comparison between groups analysis of variance. *p < 0.05, **p < 0.01.

## Result

### Expression of miR-25 in Normal Gastric Epithelial Cells and Gastric Cancer Cells

**FIGURE 1:**
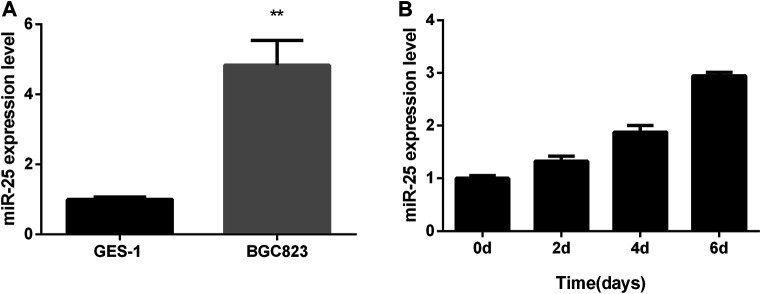
The expression profile of miR-25 in BGC823 cell. **(A)** The expression level of miR-25 was detected in BGC823 cell and GES-1 cell by RT-qPCR. **(B)** In different stages (0, 2, 4, 6 days) of culture, miR-25 expression level was detected by RT-qPCR. Value in graphs represents means ± SD. At least three independent experiments were carried out. **p < 0.01.

Total RNA from *GES-1* cells and *BGC823* cells were extracted at the growth stage, respectively. Real-time quantitative PCR (RT-qPCR) result showed that the expression level of miR-25 in *BGC823* cells was significantly higher than that in *GES-1* cell (Figure 1A). Further RT-qPCR results showed that the expression of miR-25 in *BGC823* gastric cancer cells at the growth stage was found to have an upward trend (Figure 1B). All these results indicated that the expression characteristic of miR-25 might be related to the occurrence of gastric cancer.

### miR-25 Promotes Gastric Cancer Cells Growth

**FIGURE 2:**
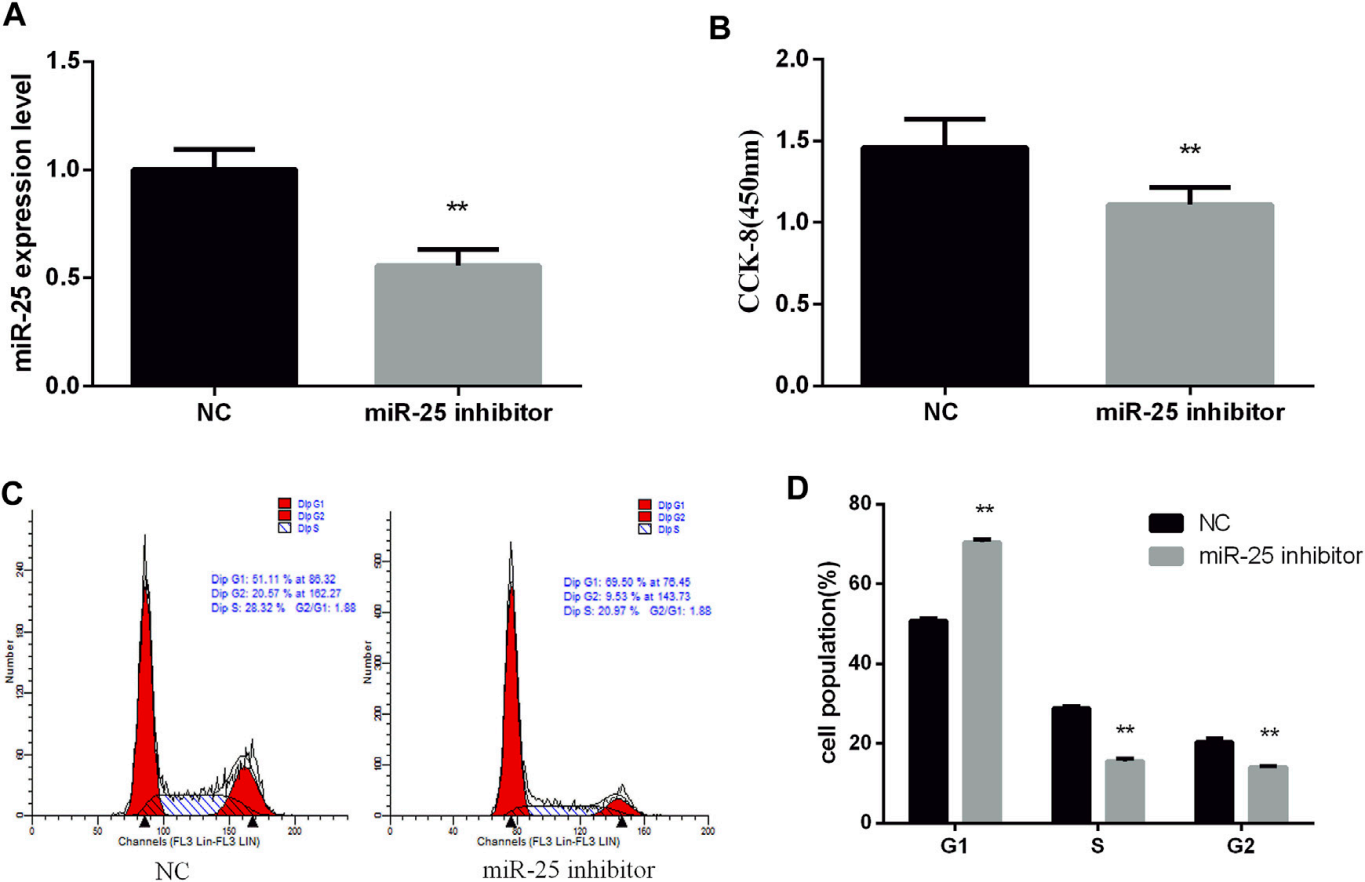
Inhibition of miR-25 inhibits BGC823 cell growth. **(A)** After transfection of miR-25 inhibitor into BGC823 cell, the expression level of miR-25 was detected by RT-qPCR. **(B)** After loss of miR-25, CCK-8 reagent was used to detect cell proliferation activity. **(C)** Cell cycle were analyzed by flow cytometry after being transfected with miR-25 inhibitor. **(D)** Statistical results of flow cytometry. Value in graphs represents means ± SD. At least three independent experiments were carried out. **p < 0.01.

**FIGURE 3:**
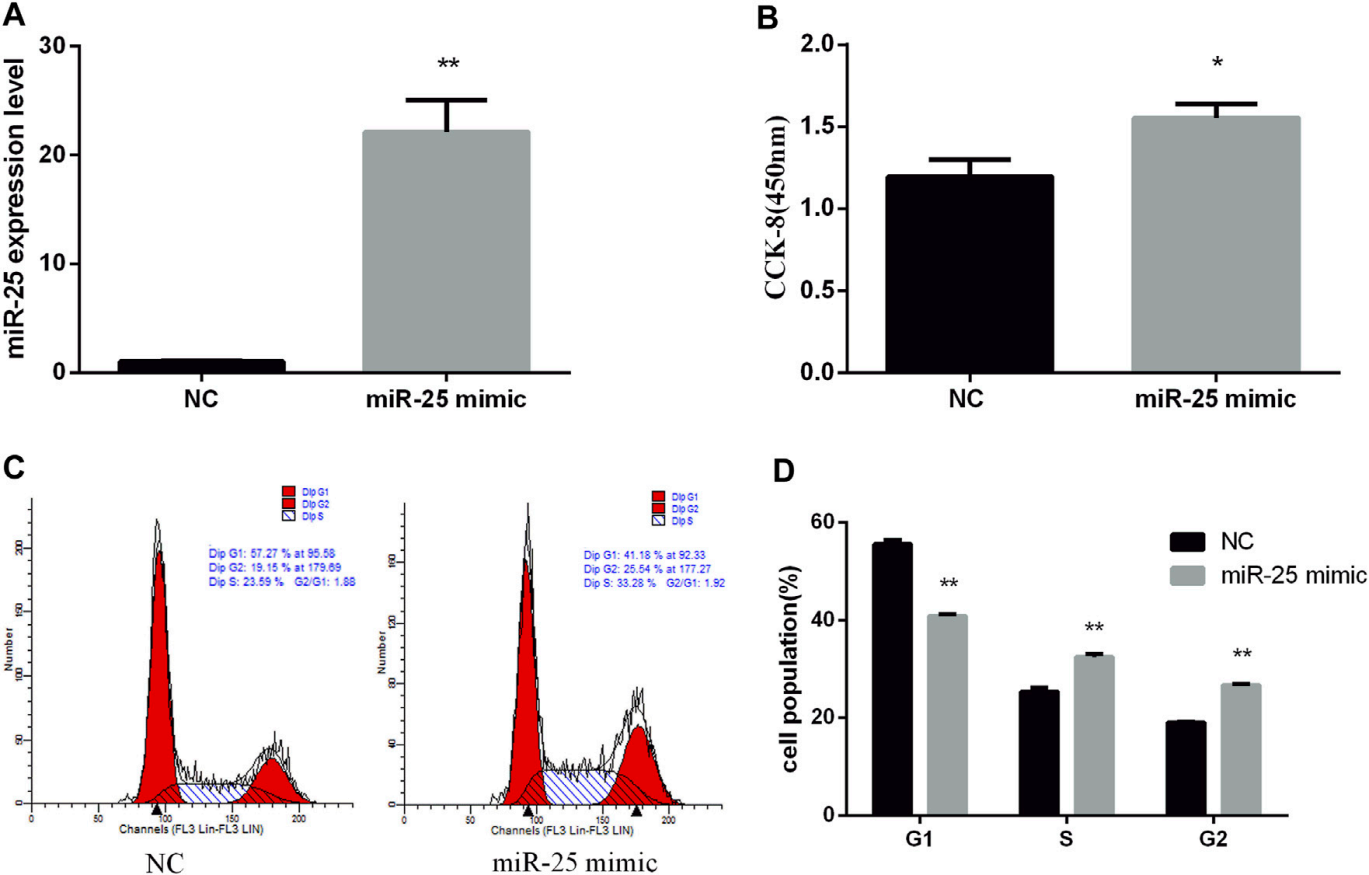
Overexpression of miR-25 promotes BGC823 cell growth. **(A)** After transfection of miR-25 mimic into BGC823 cell, the expression level of miR-25 was detected by RT-qPCR. **(B)** After gain of miR-25, CCK-8 reagent was used to detect cell proliferation activity. **(C)** Cell cycle were analyzed by flow cytometry after being transfected with miR-25 mimic. **(D)** Cell number statistics at each stage of cell cycle. Value in graphs represents means ± SD. At least three independent experiments were carried out. **p < 0.01; *p < 0.05.

To reveal the function of endogenous miR-25 for BGC823 gastric cancer cells’ growth, we transfected miR-25 inhibitor into cells to affect its expression level. RT-qPCR result showed that the expression level of miR-25 was significantly decreased compared to negative control group (Figure 2A). The proliferation activity of BGC823 cells was significantly attenuated by CCK-8 detection (Figure 2B). Flow cytometry detection of cell cycle showed that G_1_ phase cell number increased and S phase cell number decreased (Figures 2C,D). Meanwhile, we also revealed the effect of exogenous miR-25 on BGC823 cell proliferation. MiR-25 mimic was transfected into BGC823 cell, which could enhance the expression level of miR-25 (Figure 3A). Compared to the control group, overexpression of miR-25 enhanced the BGC823 cell activity by CCK-8 assay (Figure 3B). In addition, overexpression of miR-25 resulted in G_1_ phase cell population reducing and S phase cell population increasing (Figures 3C,D). Together, these data revealed that miR-25 could promote BGC823 gastric cancer cells’ growth.

### miR-25 Inhibits Gastric Cancer Cells Apoptosis

**FIGURE 4:**
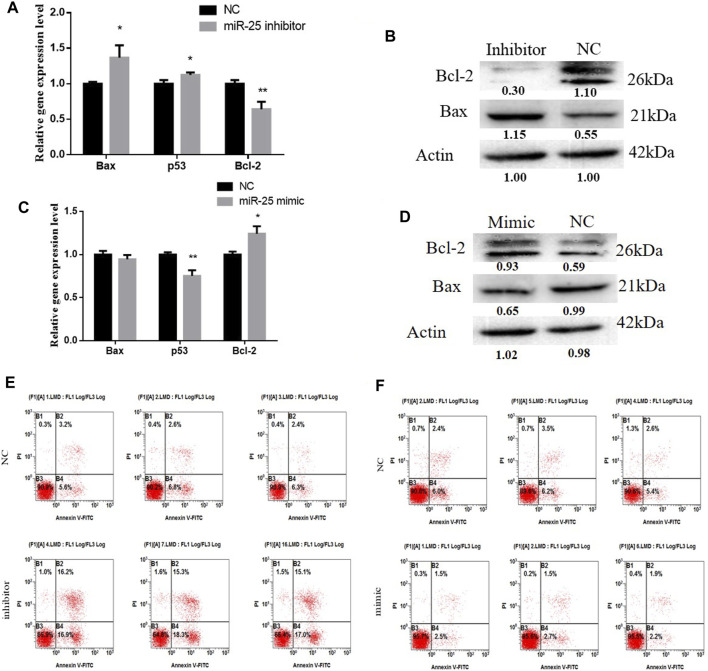
The function of miR-25 for BGC823 cell apoptosis. **(A)** After loss of miR-25, genes (Bax, p53, Bcl-2) associated with apoptosis were detected by RT-qPCR. **(B)** After loss of miR-25, Bax and Bcl-2 protein level were detected by western blot. **(C)** After gain of miR-25, genes (Bax, p53, Bcl-2) associated with apoptosis were detected by RT-qPCR. **(D)** After gain of miR-25, Bax and Bcl-2 protein level were detected by western blot. **(E)** After loss of miR-25, cell apoptosis was detected by Annexin V-FITC reagent. **(F)** After gain of miR-25, cell apoptosis was detected by Annexin V-FITC reagent. Value in graphs represents means ± SD. At least three independent experiments were carried out. **p < 0.01; *p < 0.05.

The function of miRNAs in the apoptosis of gastric cancer cells might provide an effective way to treat tumorigenesis. To elucidate the role of miR-25 for BGC823 cell apoptosis, we used molecular biology techniques and cell biology techniques to detect its effects. Firstly, after transfection of miR-25 mimic or inhibitor into BGC823 cell, we used RT-qPCR to detect the mRNA expression level of Bax, Bcl-2, and p53. Compared to the NC group, knockdown of miR-25 enhanced Bax and p53 expression level, but impeded Bcl-2 expression level (Figure 4A). The western blot analysis results indicated that knockdown of miR-25 enhanced Bax expression level and reduced Bcl-2 expression (Figure 4B). By contrast, overexpression of miR-25 reduced the Bax and p53 expression level, but induced Bcl-2 expression level (Figure 4C). Bax protein level was decreased and Bcl-2 protein level was increased (Figure 4D). Then, we used Annexin V-FITC/PI dual-staining apoptosis detection kit to detect gastric cancer cells’ apoptosis after gain or loss of miR-25. The result revealed that knockdown of miR-25 induced the number of gastric cancer cells’ apoptosis (Figure 4E). Meanwhile, overexpression of miR-25 reduced the number of apoptotic cells (Figure 4F). Together, these results demonstrated that miR-25 could inhibit gastric cancer cells’ apoptosis.

### EGR2 is a Target Gene of miR-25 in Gastric Cancer Cells

**FIGURE 5:**
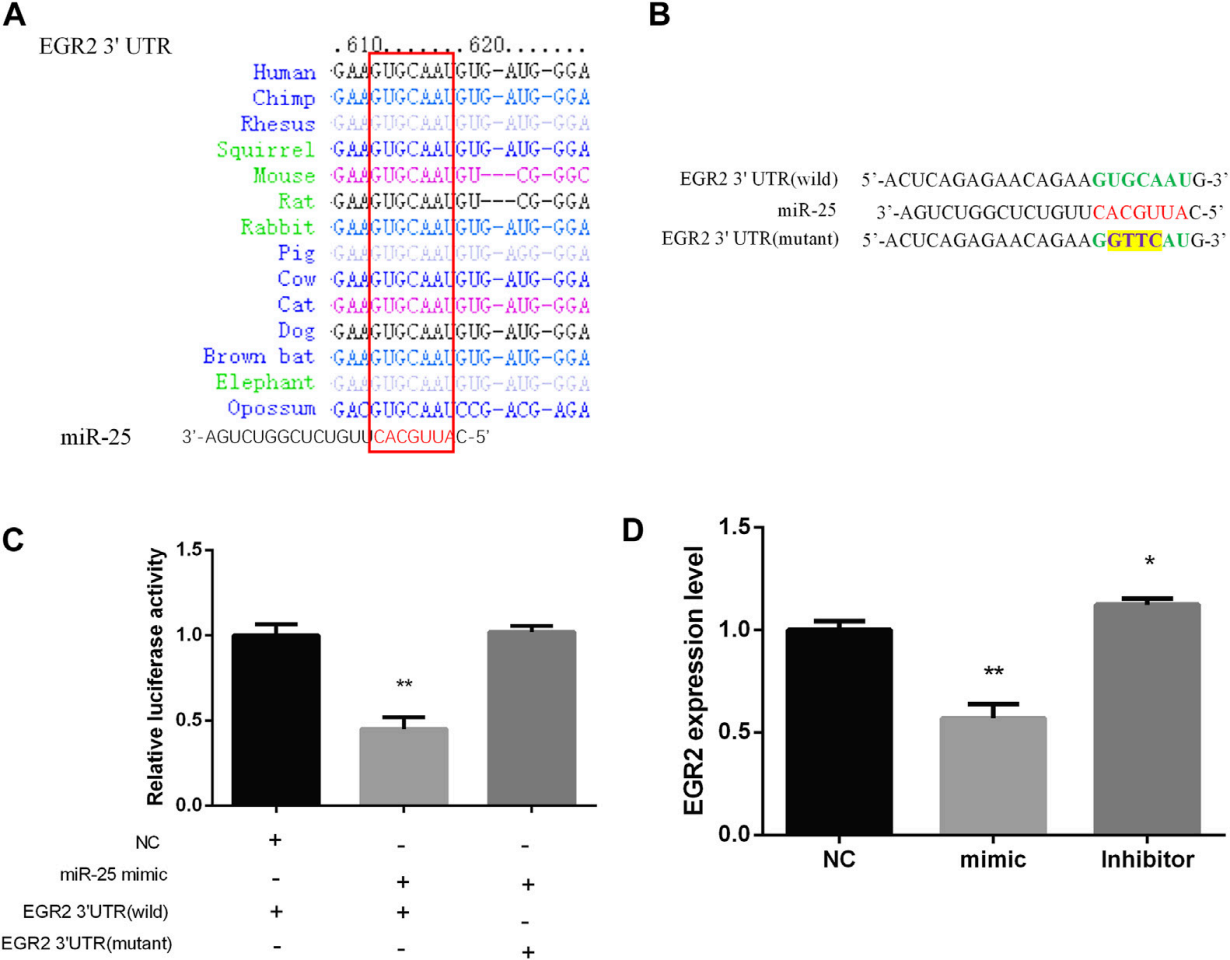
EGR2 is a target gene of miR-25. **(A)** miR-25 is highly conserved in various species. **(B)** Dual-luciferase reporter vectors containing the 3′UTR sequence of EGR2 gene (wild type and mutant type), respectively. **(C)** Luciferase activity analysis was detected after co-transfection miR-25 and dual-luciferase reporter vectors into cell. **(D)** EGR2 mRNA level was detected after gain or loss miR-25. Value in graphs represents means ± SD. At least three independent experiments were carried out. **p < 0.01; *p < 0.05.

Bioinformatics methods and a series of experiments were used to screen and verify target genes of miR-25. EGR2 caught our attention by Targetscan software prediction, the 3 ′UTR sequence of EGR2 is completely complementary to the seed region sequence of miR-25, and is also highly conserved in various species (Figure 5A). In order to identify the targeting relationship between miR-25 and EGR2, we constructed dual-luciferase reporter vectors containing the 3′UTR sequence of EGR2 gene (wild type and mutant type) respectively (Figure 5B). Firstly, miR-25 mimic was co-transfected with a dual-luciferase reporter vector containing the 3′UTR sequence of EGR2 gene (wild type or mutant type). The result revealed that overexpression of miR-25 reduced the luciferase activity of EGR2 gene 3 ′UTR wild-type reporter vector, but it did not affect the mutant vector activity (Figure 5C). Moreover, we also detected EGR2 mRNA level by RT-qPCR after gain or loss of miR-25. The results indicated that overexpression of miR-25 reduced EGR2 mRNA expression level, but knockdown of miR-25 enhanced EGR2 mRNA expression level (Figure 5D). These results revealed that EGR2 was a target gene of miR-25 in gastric cancer cells.

### Knockdown of EGR2 Counteracts the Role of miR-25 Inhibitor

**FIGURE 6:**
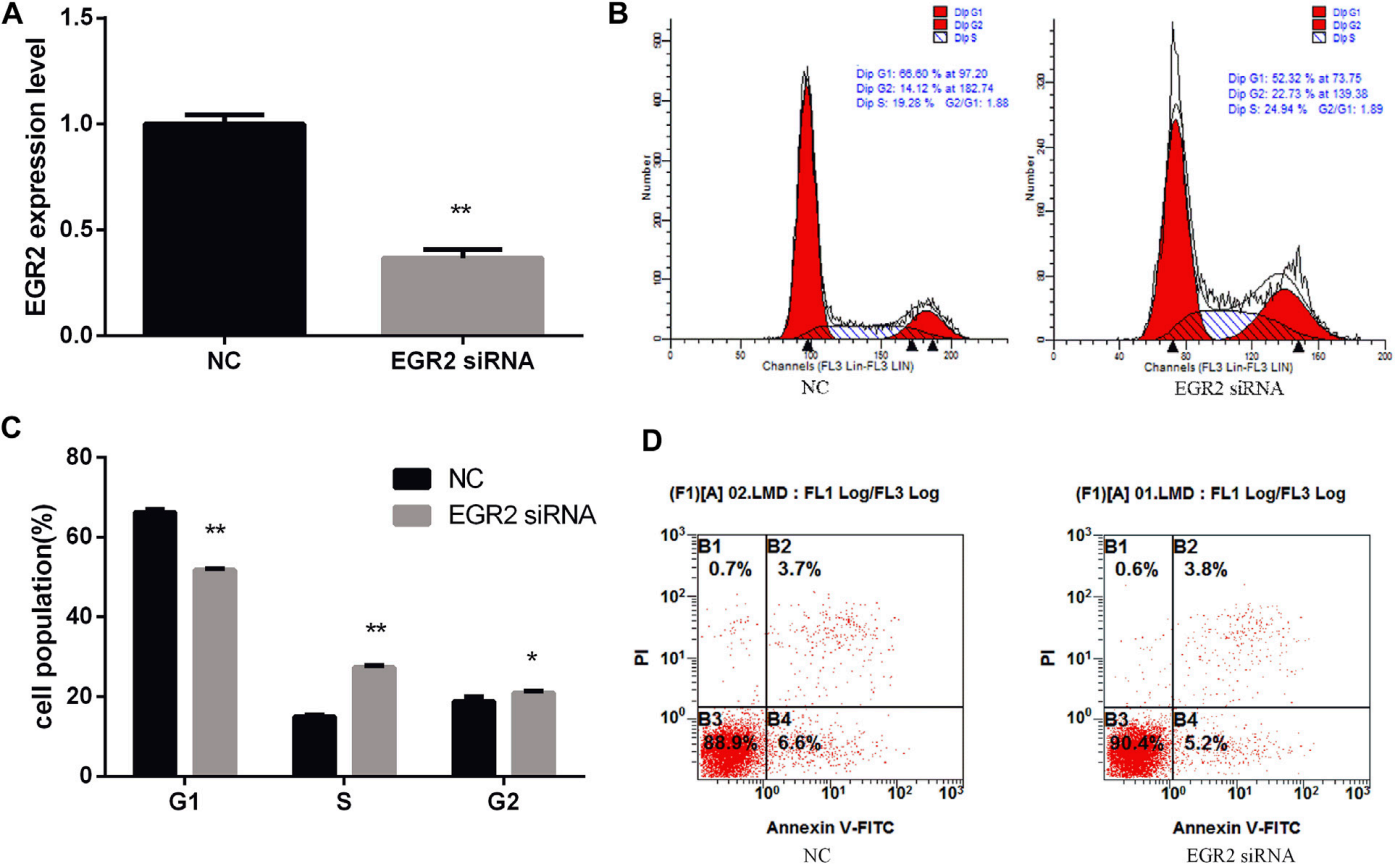
Knockdown of EGR2 promotes BGC823 cell growth and inhibits cell apoptosis. **(A)** After knockdown of EGR2, the expression level of EGR2 was detected by RT-qPCR. **(B)** Cell cycle were analyzed by flow cytometry after being transfected with EGR2 siRNA. **(C)** Cell number statistics at each stage of cell cycle. **(D)** After knockdown of EGR2, cell apoptosis was detected by Annexin V-FITC reagent. Value in graphs represents means ± SD. At least three independent experiments were carried out. **p < 0.01; *p < 0.05.

**FIGURE 7:**
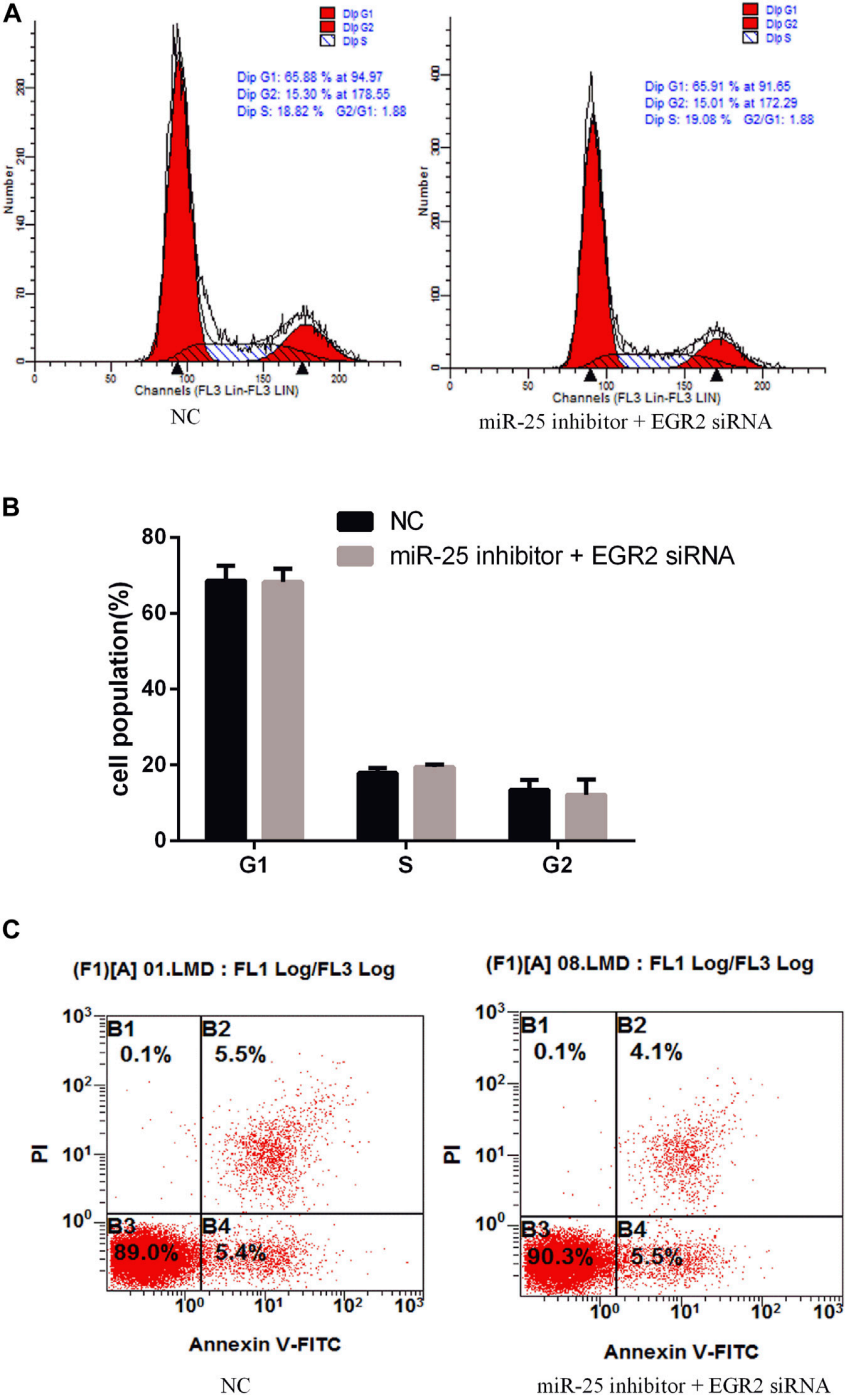
Knockdown of EGR2 counteracts the role of miR-25 inhibitor. **(A)** Cell cycle were analyzed by flow cytometry after being co-transfected with miR-25 inhibitor and EGR2 siRNA. **(B)** Cell number statistics at each stage of cell cycle. **(C)** After being co-transfected miR-25 inhibitor and EGR2 siRNA, cell apoptosis was detected by Annexin V-FITC reagent.

To investigate the function of EGR2 in gastric cancer cells’ growth and apoptosis, we used EGR2 siRNA to reduce the expression of EGR2 (Figure 6A). First, the cell cycle assay results indicated that knockdown of EGR2 reduced the number of G1 phase and elevated the S phase cell population (Figures 6B,C). Meanwhile, we also detected the number of apoptosis cells’ changes by Annexin V-FITC/PI dual-staining apoptosis detection kit. The result showed that knockdown of EGR2 could inhibit gastric cancer cells’ apoptosis (Figure 6D). Together, these results demonstrated that knockdown of EGR2 promotes gastric cancer cells’ growth and inhibits their apoptosis. To further reveal the relationship of miR-25 and EGR2, an antagonistic experiment was designed. miR-25 inhibitor and EGR2 siRNA were co-transfected into gastric cancer cells. The cell cycle assay results revealed that there was no significant change in the number of cells at each stage (Figures 7A,B). Compared to the control group, there was also no significant difference in the number of apoptosis cells (Figure 7C). These data demonstrated that Knockdown of EGR2 counteracts the role of miR-25 inhibitor in gastric cancer cells.

## Discussion

In the present study, we found a differentially expressed miRNA in BGC823 gastric cancer cells and normal gastric mucosa cell (GES-1 cells and RGM-1 cells): miR-25. Overexpression of miR-25 promoted BGC823 gastric cancer cells and inhibited their apoptosis. EGR2 was a target gene of miR-25. Knockdown of EGR2 could promote gastric cancer cells’ growth and inhibit their apoptosis. Therefore, miR-25 might be used as a potential biomarker or therapeutic target for gastric cancer.

MiRNAs are a class of widely distributed endogenous non-coding RNA, with a size of 20–25 nucleotide, which are highly conserved genetically and can negatively or positively regulate the expression of their target genes (Gambari et al., 2011). Studies have found that miRNAs can be used as oncogenes or tumor suppressor genes to participate in the regulation of cell growth, apoptosis, and cycle (Voorhoeve et al., 2006; Manikandan et al., 2008). Some miRNAs are directly involved in the formation of human tumors (such as lung cancer, breast cancer, craniocerebral tumor, liver cancer, colorectal cancer, and lymphoma). MiRNAs can be used as both oncogenes and tumor suppressor genes to participate in multiple signaling pathways of human tumor formation (Esquela-Kerscher and Slack, 2006). Therefore, the study of specific miRNA function provides a new direction for tumor treatment and prevention.

The role of miR-25 in the pathogenesis of malignant tumors has been studied frequently. In human ovarian cancer, up-regulation of miR-25 could enhance cell proliferation and down-regulation of miR-25 could enhance the expression of pro-apoptotic proteins such as Bax and caspase-3 (Zhang et al., 2012). In non-small cell lung cancer (NSCLC), the expression level of miR-25 was up-regulated. Over-expression of miR-25 could significantly increase NSCLC cells’ proliferation, migration, and invasion, but down-regulation of miR-25 remarkably reduced cell proliferation, migration, and invasion in NSCLC cells. Further results demonstrated that FBXW7 was a direct target gene of miR-25 (Xiang et al., 2015). Moreover, in gastric cancer, overexpression of miR-25 could promote gastric cancer cell proliferation, migration, and invasion via targeting RECK (Zhao et al., 2014). This is consistent with our current findings.

In conclusion, we found that a high expression of miR-25 in BGC823 gastric cancer cells can enhance cell growth and reduce cell apoptosis via targeting gene *EGR2*. The result revealed that miR-25 might be a potential therapeutic target for gastric cancer.

## Data Availability

The original contributions presented in the study are included in the article/Supplementary Material, further inquiries can be directed to the corresponding author.
